# Ex‐vivo investigation of radiofrequency ablation in pancreatic adenocarcinoma after neoadjuvant chemotherapy

**DOI:** 10.1002/deo2.152

**Published:** 2022-07-14

**Authors:** Gemma Rossi, Maria Chiara Petrone, Marco Schiavo Lena, Luca Albarello, Diego Palumbo, Sabrina Gloria Giulia Testoni, Livia Archibugi, Matteo Tacelli, Piera Zaccari, Giuseppe Vanella, Laura Apadula, Stefano Crippa, Giulio Belfiori, Michele Reni, Massimo Falconi, Claudio Doglioni, Francesco De Cobelli, Andrew J Healey, Gabriele Capurso, Paolo Giorgio Arcidiacono

**Affiliations:** ^1^ Division of Pancreato‐Biliary Endoscopy and Endosonography, Pancreas Translational and Clinical Research Center, San Raffaele Scientific Institute IRCCS Vita Salute San Raffaele University Milan Italy; ^2^ Division of Pathology, Pancreas Translational and Clinical Research Center, San Raffaele Scientific Institute IRCCS Vita Salute San Raffaele University Milan Italy; ^3^ Department of Radiology Pancreas Translational and Clinical Research Center San Raffaele Scientific Institute IRCCS Vita Salute San Raffaele University Milan Italy; ^4^ Division of Pancreatic Surgery, Pancreas Translational and Clinical Research Center, San Raffaele Scientific Institute IRCCS Vita Salute San Raffaele University Milan Italy; ^5^ Division of Oncology, Pancreas Translational and Clinical Research Center, San Raffaele Scientific Institute IRCCS Vita Salute San Raffaele University Milan Italy; ^6^ Department of Clinical Surgery Royal Infirmary of Edinburgh, University of Edinburgh Edinburgh UK

**Keywords:** endoscopic ultrasound, ex‐vivo, neoadjuvant chemotherapy, pancreatic adenocarcinoma, radiofrequency ablation

## Abstract

**Objective:**

Endoscopic ultrasound (US)‐guided radiofrequency ablation (RFA) has been investigated for pancreatic ductal adenocarcinoma (PDAC) but studies are limited and heterogeneous. Computed tomography (CT) scan features may predict RFA response after chemotherapy but their role is unexplored. The primary aim was to investigate the efficacy of ex‐vivo application of a dedicated RFA system at three power on surgically resected PDAC in patients who underwent neoadjuvant chemotherapy. The secondary aim was to explore the association between pre‐treatment CT‐based quantitative features and RFA response.

**Methods:**

Fifteen ex‐vivo PDAC samples were treated by RFA under US control at three power groups (10, 30, and 50 W). Short axis necrosis diameter was measured by two expert blinded pathologists as the primary outcome. Two radiologists independently reviewed preoperative CT images.

**Results:**

Eighty percent of specimens showed coagulative necrosis consisting of few millimeters: 5.7 ± 3.9 mm at 10 W, 3.7 ± 2.2 mm at 30 W, and 3.5 ± 2.4 mm at 50 W (*p* = 0.3), without a significant correlation between power setting and mean necrosis short axis (rho = –0.28; *p* = 0.30). Good agreement was seen between pathologists (*k* = 0.76; 95% confidence interval 0.55–0.98). Logistic regression analysis did not show associations between CT features and RFA response.

**Conclusions:**

RFA causes histologically evident damage with coagulative necrosis of a few millimeters in 80% of ex‐vivo PDAC samples after chemotherapy and no clinical or pre‐operative CT features can predict efficacy. Power settings do not correlate with the histological ablation area. These results are of relevance when employing RFA in vivo and planning clinical trials on its role in PDAC patients.

## INTRODUCTION

Pancreatic ductal adenocarcinoma (PDAC) has a dismal prognosis, with a 5‐year survival rate of <10%.^1^ A multimodal approach with the addition of chemotherapy to radical surgical resection is a potentially curative treatment, but only 15%–20% of patients are eligible for surgery at diagnosis,^2^ as the majority present with either metastatic or locally advanced unresectable disease.^3^ Despite new intensified chemotherapy regimens are now available, they only lead to marginally increased survival in the advanced disease setting.^4^

Radiofrequency ablation (RFA) under endoscopic ultrasound (EUS) control is gaining more interest as a locoregional minimally invasive technique, allowing neoplastic tissue ablation by inducing thermal coagulation. RFA has been successfully applied to different kinds of neoplasms;^5^ however, its application to the pancreas is considered with caution, due to potential damage to surrounding structures and the pancreas itself.^6^

RFA probes are designed to obtain a spherical ablation area and area treatment width is influenced by the thermal efficiency of the delivery system and by intrinsic tissue factors.^7^, ^8^

EUS‐guided RFA systems use has been investigated both in pre‐clinical animal models^9^, ^10^ and clinical settings for pancreatic masses,^11^, ^12^, ^13^ but these studies showed heterogeneity in terms of methodology, ablation settings, and results.^6^, ^14^ EUS‐RFA was applied in locally advanced PDAC with different ablation powers and post‐ablative protocols to control coagulative necrosis obtained within the lesion, with promising results.^15^, ^16^, ^17^

Thus, whether applied power influences coagulative necrosis size/area is unclear. This holds particular importance for locally advanced and recently also borderline resectable/resectable PDAC previously treated with chemotherapy, where tissue is particularly fibrotic and/or necrotic. In this view, the use of computed tomography (CT) scans quantitative features reflecting fibrotic and necrotic components of such lesions after chemotherapy may be an appealing tool to predict RFA response. Indeed, CT features have been employed not only to assess RFA response in other tumors^18^ but also to predict chemotherapy response in advanced PDAC.^19^

The primary study aim was to investigate the ex‐vivo effect in terms of coagulative necrosis of a dedicated RFA system at three different ablation powers on surgically resected specimens of PDAC patients who underwent neoadjuvant chemotherapy. The secondary aim was to explore the association between pre‐treatment radiological features and RFA response.

## METHODS

The study protocol was approved by Scientific Institute San Raffaele institutional review board (number: RFA‐ex vivo 2016). Patients signed informed consent. The study was conducted between September 2019 and June 2020. Inclusion criteria were patients with resected PDAC who had undergone neoadjuvant chemotherapy before surgery and with a primary lesion of at least 2 cm in size measured at preoperative CT imaging. Patient demographics and clinical features were recorded in a dedicated database.^20^

Preoperatively (within 14 days) contrast‐enhanced CT scan was performed on all patients to confirm resectability. CT protocol included administration of intravenous non‐ionic iodine contrast medium and consisted of a multiphase acquisition (unenhanced, late arterial and portal venous abdomen axial scans; late phase was also routinely performed).^21^ Two experienced radiologists (Diego Palumbo and Francesco De Cobelli) independently reviewed images, blinded to ablation setting and outcome. Selected CT findings were both qualitative (necrosis and late enhancement) and quantitative (Hounsfield unit [HU] assessment measured by means of standardized regions of interest in each phase [unenhanced, late arterial, portal venous, and late phase] at the same level of index lesion). The difference between HU measured in the portal and unenhanced phase was considered a surrogate of necrosis,^22^ whereas the difference between HU in the late and unenhanced phases was considered a surrogate of lesion fibrous content.^23^

Immediately after resection, fresh pancreatic specimens were collected from the operating room and transported to an endoscopy suite. Specimens were evaluated together by a gastrointestinal sonographer and pathologist, in order to preserve tissue from burning for a postoperative definitive histopathological report. Specimens were positioned on a bowl containing scant water, connected by grounding plates to an RFA generator, and were treated at different powers under ultrasound (US) guidance by using an external 7.5‐ to 10‐MHz US probe (Figure S1).

RFA system employed (STARmed Co. Ltd, Koyang, Korea) is designed to permit a mini‐invasive in‐vivo pancreatic lesion ablation under EUS‐control consisting of a radiofrequency generator (VIVA generator) delivering energy connected to the needle (similar to EUS needles) with a specific monopolar electrode positioned on the distal tip (Figure S2). A peristaltic pump connected to the needle allows continuous chilled saline solution perfusion to avoid tissue charring. Power is set on the generator and the system can automatically modulate and decrease it if tissue impedance quickly increases during ablation. Ex‐vivo RFA was performed on the basis of previous ex‐vivo‐animal tests^24^ by using a US probe to establish the central point to position the electrode. On the basis of previous experience, a 19 Gauge needle (EUSRA) was used with a 1 cm electrode length in order to standardize the method.

Three different powers were set on a generator and applied in three specimen groups: five were treated at 50 W, five at 30 W, and five at 10 W. A randomization list was used to allocate 15 surgical specimens to one of three ablation power groups. Ablation time was dependent on tissue impedance and the system was stopped if impedance quickly raised beyond safety thresholds (impedance ≥ 500 Ohm and tissue temperature >100°C). It was, however, established to stop treatment after 120 s in all cases. During RFA a hyperechoic area appears around the distal tip of US. At the ablation end, the US ablation area diameter was registered and before needle removal, an additional needle was positioned within the tumor, recapitulating the RFA needle trajectory (landmark for pathologists). Subsequently, specimens were transferred to the pathology unit, fixed in 10% buffered formalin, and sampled according to internal protocol.

On the day after the procedure, specimens were sampled by two blinded (about power assignment) expert pancreatic pathologists (Marco Schiavo Lena and Luca Albarello). Formalin‐fixed and paraffin‐embedded blocks of the whole tumor bed and adjacent parenchyma were cut into 4–5 mm slides. At the microscopic examination, coagulative necrosis presence was recorded and expressed as the diameter around the probe insertion point into neoplastic tissue. The mean of two pathologists' measurements was used in the final analysis. The presence of microscopic air bubbles around necrotic tissue was recorded in a dichotomic way: present/absent.

Given the exploratory nature of the study, no power calculation was performed ahead. Data are presented as the mean (±SD) for normally distributed variables and the median (interquartile range [IQR]) for a skewed distribution and are compared by means of a one‐way analysis of variance test for normally distributed variables and Kruskal–Wallis test for skewed ones. Correlation between continuous variables was calculated with the Pearson correlation test. Pathologists' agreement in defining the short‐axis diameter of the ablated area was measured by means of K agreement.

In order to identify CT scan features associated with better treatment response, samples were classified as having a good response if the mean diameter of the ablated area was in the highest 50th percentile. A receiver operating characteristic curve was then plotted to determine the optimal cutoff value of either necrosis or fibrous content to predict a good response. *p*‐values were considered significant when <0.05. Statistical analysis was performed using dedicated software (Medcalc 12.1, Belgium). Logistic regression analysis was planned, in order to identify factors associated with a good treatment response.

## RESULTS

Fifteen patients (mean age 63 ± 11 years, 53% males) who underwent pancreatic resection for PDAC after neoadjuvant chemotherapy (seven m‐FOLFIRINOX, eight gemcitabine‐based) were enrolled. The mean lesion size was 31.3 ± 10 mm at surgical pathology gross examination. Of them, 11 (73.3%) had a pancreaticoduodenectomy and four (26.7%) underwent a distal pancreatectomy.

Of 15 patients, five (33.3%) presented histological scarce chemotherapy response (Hartmann grade 0), four (26.7%) moderate response (Hartmann 1) and six (40%) good (Hartmann 2). At the final pathologist examination, 12 patients had stage IIB disease according to TNM Classification of Malignant Tumors (8th edition)^25^ and others stage I (two‐stage IB and one‐stage IA). The tumor grade was 3 in eight (53.3%), 2 in seven (46.7%) specimens. Clinical and pathological patient features are summarized in Table 1. Histology showed that the tumor diameter was significantly larger at 10 W (*p* = 0.04).

**TABLE 1:** Clinical and pathology features of the 15 patients whose pancreatic specimens were randomized to three different ablation power

	Ablation power				
	50 W	30 W	10 W	Total	*p‐*value
**Clinical features and demographics**					
Patients (*n*)	5	5	5	15	–
Male sex	3 (60%)	2 (40%) (20%)	3 (60%)	8 (53%)	0.99
Mean age (± SD)	62 (±15)	66 (±13)	61 (±7)	63 (±11)	0.4
Head resection	3 (60%)	4 (80%)	4 (80%)	11 (73%)	0.2
Mean (± SD) tumor size at pathology (mm)	27 (±7)	26 (±4)	40 (±13)	31 (±11)	0.04
Hartmann grade 2	2 (40%)	3 (60%)	1 (20%)	6 (40%)	0.4
Tumor grade					0.99
2	3	2	2	7	
3	2	3	3	8	
TNM stage					1
1	1	1	1	3	
2	4	4	4	12	
Neoadjuvant chemotherapy (m‐FOLFIRINOX)	1 (20%)	3 (60%)	3 (60%)	7 (47%)	0.4
Median (IQR) CA 19‐9 before surgery	28 (20–51)	105 (17–159)	63 (28–279)	48 (18–75)	0.5
Biliary metal stent in place at time of RFA	2 (40%)	3 (60%)	2 (40%)	7 (47%)	0.1

Abbreviations: IQR, interquartile range; RFA, radiofrequency ablation; TNM, TNM Classification of Malignant Tumors.

After surgery, samples were randomized into ablation power groups and treated accordingly by the same gastrointestinal sonographer. Ex‐vivo treatment was technically feasible in all cases. In 7/11 (63.6%) patients who underwent pancreaticoduodenectomy, a partially covered metal biliary stent was present and removed only in two patients being left in place in the remaining without RFA system malfunctions. Ablation time was significantly longer at 10 W (120 s median ablation time; IQR 112.5–120) than those observed with 30 W (median 20 s; IQR 10.7–20.2) and 50 W (median 8 s; IQR 7.7–9.2) (*p* = 0.0019 at Kruskal–Wallis test). There was a significant negative correlation between power and ablation time (rho = −0.97; *p* < 0.001).

US‐mean short‐axis diameter of necrotic tissue was 7.6 mm (±3), being respectively 11 mm (±3), 7.4 mm (±1.5), and 4.6 mm (±1.7) in groups at 10, 30, and 50 W, significantly larger in the first (*p* = 0.002).

Although US ablation was considered feasible and effective in all 15 cases, 12 (80%) specimens showed histological coagulative necrosis after RFA contained within the tumor regardless of applied power without any damage to the remaining pancreas. In the other three specimens (one patient for each power) no signs of coagulative necrosis were evident. There was good agreement between two blinded pathologists regarding ablated area measures with a weighted *k* = 0.76 (95% confidence interval 0.55–0.98). The mean short‐axis diameter (perpendicular to the needle probe) of histological coagulative necrosis in 15 patients was 5.4 mm (±2.2).

**FIGURE 1:**
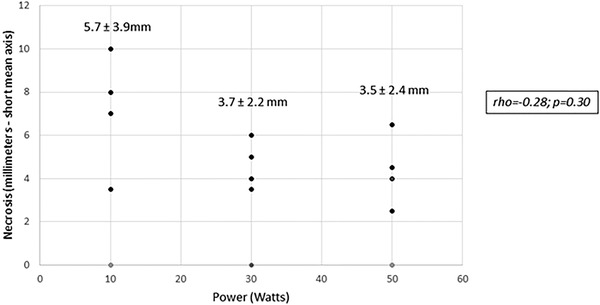
Necrosis size obtained with three different ablation powers applied on the lesions (not statistically significant mean values of necrosis at different ablation powers are reported)

**FIGURE 2:**
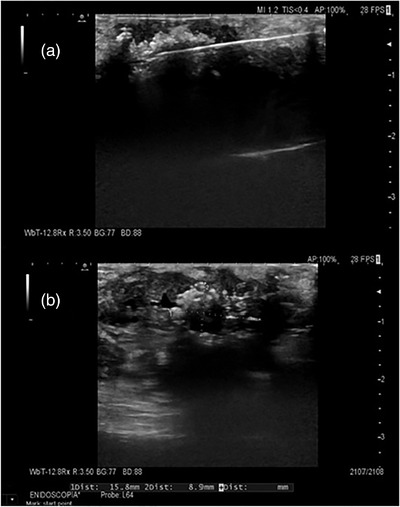
Ultrasound appearance of ablated lesions. (a) Ultrasound image during the radiofrequency ablation procedure with needle inside the lesion and hyperechoic bubbles around it as an effect. (b) Ultrasound image of the ablated area (size 15.8 × 8.9 mm^2^) inside the hypoechoic lesion represented by hyperechoic bubbles

**FIGURE 3:**
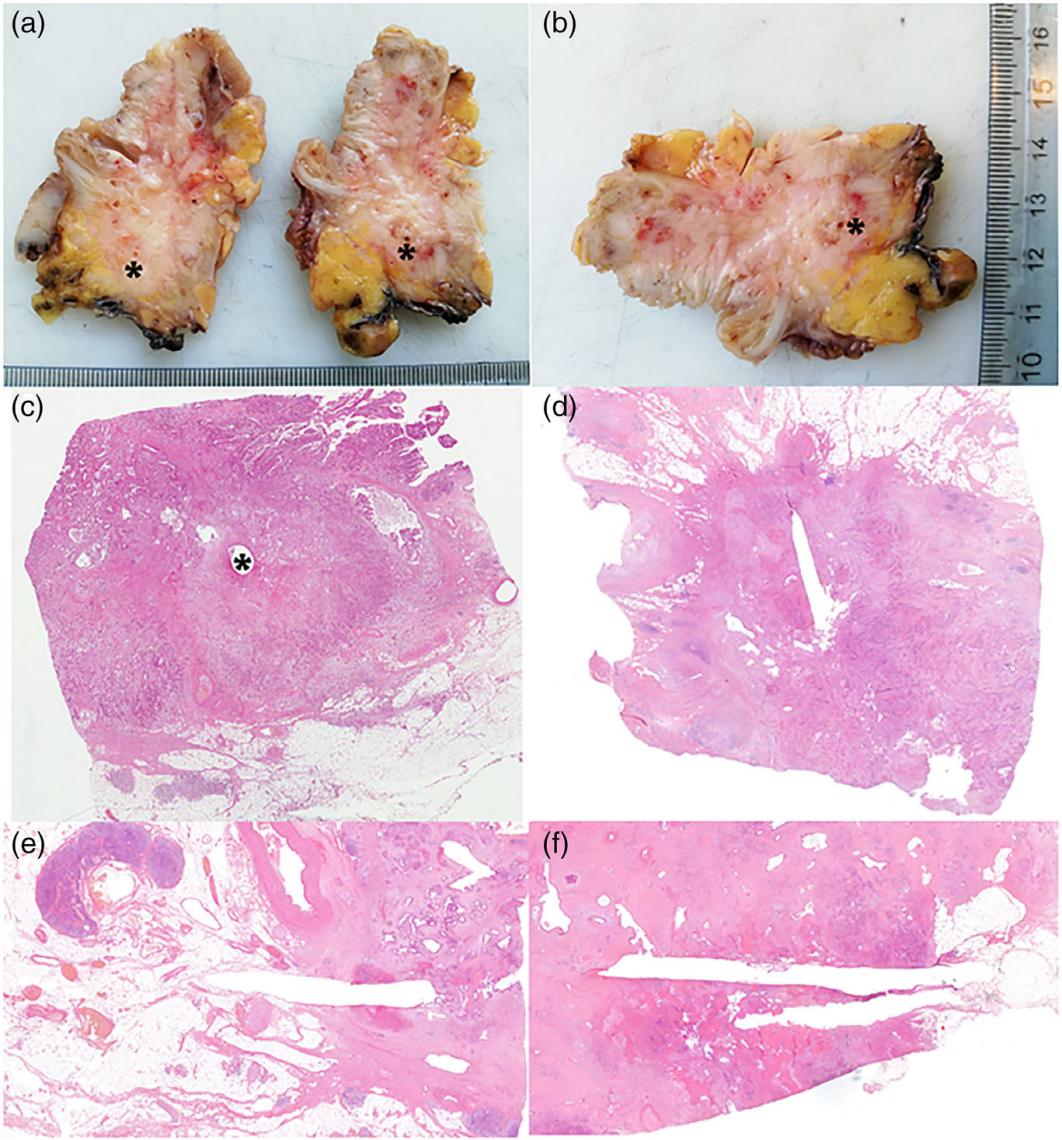
Gross pictures of a pancreaticoduodenectomy specimen after the axial cut. (a, b) The asterisk indicates the radiofrequency ablation (RFA) needle insertion points. The needle track is clearly recognizable on a histological level, both in (c) orthogonal (asterisk) and (d–f) parallel sections

**FIGURE 4:**
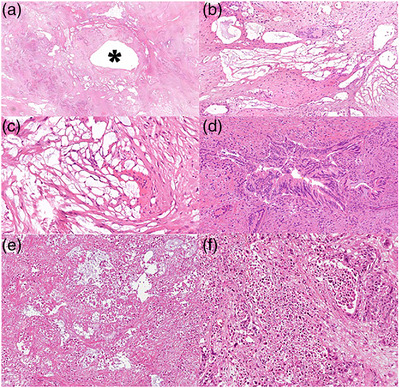
(a) The “hole” left by the needle track is in the middle of the picture (asterisk); there is an area of tissue damage around it. (b, c) Air bubbles were created by the rapid increase of temperature at higher powers and they appeared as a ring around the area of tissue damage. (d) A neoplastic gland with procedure‐induced cytological damage. The elongation of the nuclei is evident. (e, f) Neoplastic necrosis within the tissue damage area induced by the procedure

There was, instead, no correlation between the US‐short axis diameter of the necrotic area and the mean value measured by pathologists (rho = 0.13; *p* = 0.62). The histological mean short axis necrosis diameter was 5.7 ±3.9 mm at 10 W, which was slightly, but not significantly larger than the mean of 3.7 ± 2.2 mm at 30 W and of 3.5 ± 2.4 mm at 5 W (*p* = 0.3 in both cases). However, no significant correlation was seen between the power setting group and the mean short axis necrotic area diameter measured by pathologists (rho = −0.28; *p* = 0.30; Figures 1, 2, 3, 4). At the highest power (50 W) microscopic air bubbles around histological necrosis were seen by both pathologists in two of four specimens, as a ring created by rapid temperature increase.

Preoperative CT‐scan lesion features are presented in Table 2. None of the quantitative CT findings or necrosis or fibrosis parameters were different among the groups. In order to investigate whether there were CT‐scan features associated with a better RFA response, we classified 15 samples as those with a good response when the short axis size of the necrotic area was above the median value of 4 mm. There was no association between evaluated CT features and RFA response (see Table 2). We also compared HU values of three samples in which treatment was not effective and 12 with identified necrosis, without finding significant differences. A receiver operating characteristic curve analysis was run to try to identify CT features associated with a good response. However, CT features did not seem able to predict response, as an estimate of necrosis, calculated as the delta between portal‐basal HU > 33.3 was associated with a good response with a sensitivity of 85.7% but with a low specificity of 50% (area under the curve [AUC] 0.52). Similarly, an estimate of fibrosis, calculated as the delta between late‐basal HU > 39.8 was associated with a good response with 100% sensitivity but only 33.3% specificity (AUC 0.58).

**TABLE 2:** Computed tomography scan features of the 15 samples according to the three different power settings

	Ablation power				
	50 W	30 W	10 W	Total	*p‐*value (Kruskall‐Wallis)
Radiological features					
Mean basal HU	31 (20–42)	32 (17–48)	35 (29–41)	33 (29–37)	0.7
Portal phase HU	76 (60–93)	70 (26–114)	84 (65–103)	77 (64–89)	0.6
Late phase HU	96 (65–127)	86 (30–141)	94 (77–112)	91 (74–108)	0.9
Necrosis (portal‐basal)	44 (26–63)	40 (−13–93)	49 (33–65)	45 (33–57)	0.8
Fibrosis (late‐basal)	64 (38–90)	58 (−8–123)	61 (42–79)	61 (45–76)	0.9
EUS features before RFA					
Mean long axis (mm)	24 (22–27)	26 (17–35)	32 (28–35)	27 (24–30)	0.06

Abbreviations: EUS, endoscopic ultrasound; HU, Hounsfield unit; RFA, radiofrequency ablation.

**TABLE 3:** Logistic regression analysis of factors associated with a good response to radiofrequency ablation, defined as a necrosis area >4 mm

Factor	Necrosis area ≤ 4 mm (*n* = 8)	Necrosis area > 4 mm (*n* = 7)	OR (95% CI); *p*‐value
Mean age	61 (±14)	65.5 (±7)	1.04 (0.94–1.15); 0.41
Male sex	5 (62%)	3 (43%)	0.75 (0.09–5.7); 0.72
Mean tumor size at pathology	29 (±10)	31 (±11)	1.02 (0.92–1.12); 0.65
Hartmann grade 2	2 (25%)	4 (57%)	0.25 (0.02–2.23); 0.21
Mean CA 19‐9	65 (±79)	114 (±208)	1.00 (0.99–1.00); 0.44
Gemcitabine‐based chemo	5 (62%)	3 (43%)	0.45 (0.05–3.5); 0.44
Median pre‐treatment necrosis at CT scan (portal‐basal δHU)	42.1 (IQR 27.3–65)	47.3 (IQR 41.3–56.8)	1.00 (0.95–1.06); 0.78
Median pre‐treatment fibrosis at CT scan (late‐basal δHU)	61.8 (IQR 39.7–76)	67.2 (IQR 49.3–76.2)	1.01 (0.96–1.06); 0.70

Abbreviations: CT, computed tomography; HU, Hounsfield unit.

In logistic regression analysis (Table 3), none of the demographic, clinical, radiologic, or ablation features were significantly associated with a good RFA response.

## DISCUSSION

This is the first ex‐vivo investigation of US‐guided RFA system effectiveness in PDAC samples after neoadjuvant chemotherapy with findings of technical feasibility in 100% of cases but actual efficacy in the 80%, in absence of thermal damage to the surrounding pancreas. In addition, pre‐operative radiological features were not associated with the RFA response.

Comparing the three power settings, no significant difference was identified in ablation efficacy. Randomization of 15 surgical samples to three treatment groups allowed a good balance in terms of demographics, radiological and clinical features, apart from a larger diameter in 10 W group lesions. Coagulative necrosis obtained consisted of a few millimeters (15 specimens median necrosis: 4 mm) independent of the applied power. We specifically investigated whether different power application in the three groups induced different amounts of necrosis: a slightly larger necrotic axis was reported at 10 W for a long time but this difference did not reach statistical significance. The application time differed between groups and was significantly longer at 10 W. This is not surprising, as at higher powers, rapid temperature increases and consequently tissue impedance leads to automatic treatment interruption. Conversely, the 10 W system was stopped after a standardized time of 120 s in all cases except one (90 seconds), due to the creation of a “hyperechoic cloud” on US imaging (not necessarily corresponding to necrotic tissue at histology) that covered the entire tumor, in order to preserve histological examination.

The total lack of induced necrosis in one sample in each power group was not predictable by clinical or radiological features and not recognizable during US procedure, probably due to intrinsic characteristics of tissue previously treated by chemotherapy. In this view, the present ex‐vivo investigation offers a previously unreported and unique opportunity to evaluate histologically induced tissue damage on fresh surgical samples.

Previous studies on animal models reported variable success rates in terms of coagulative necrosis. Kim et al.^9^ described the first attempt of this RFA system standardization, treating in‐vivo normal porcine pancreas at 50 W, obtaining a well‐demarcated “ablated area” consisting of 23.0 ± 6.9 mm at gross specimen examination, with coagulative necrosis core <10 mm. Another in‐vivo study^10^ on pigs obtained similar necrosis areas, with necrosis diameter less than 1 cm (8–10 mm) on histology, surrounded by fibrotic tissue.

Compared to previous animal model findings, in the current study the necrosis area consisted of a few millimeters. This may be due to a more fibrotic tissue intrinsic to the histological nature of PDAC, especially after chemotherapy. For this reason, it is likely that more than one RFA application within the same lesion during the same treatment may be necessary to cover the entire lesion.

Standardization of the optimal ablation power setting was an aim of the study. The largest coagulative necrosis size was observed at lower RFA power (10 W), with a negative trend between powers and necrosis area (7.12 ± 2.72 mm maximum diameter at 10 W). However, this difference was not statistically significant, possibly due to small subgroups. To the best of our knowledge, there are no similar published data, either in pre‐clinical or clinical studies, on different power settings in tumors previously treated with chemotherapy.

Song et al.^15^ applied in‐vivo EUS‐guided RFA system on six locally advanced or metastatic PDAC at 20–50 W, with multiple RFA applications to cover the entire lesion, but no precise ablated area sizes were reported. Crinò et al.^16^ treated seven locally advanced PDAC at 30 W, with multiple applications if necessary on the basis of tumor size. The mean “thermal damage volume” was estimated at CT at one day and one month being of 3.75 cm^3^ (range 0.72–12.6 cm^3^) corresponding to 30% of the tumor mass. Scopelliti et al.^17^ treated 10 unresectable non‐metastatic PDAC with EUS‐RFA at 20 or 30 W for a time depending on tissue impedance eventually using multiple passages. A “hypodense intra‐tumor area” consisting of a 30 mm mean diameter was seen in all patients at 30‐day CT scan.

Of note, unlike the present study, none of the previous studies performed histopathological evaluations. Thus, the actual histopathological RFA effect was largely unknown and this experience could offer a possibility of necrotic damage measurement on cancers previously treated by chemotherapy. Moreover, chemotherapy is now increasingly applied to resectable or borderline resectable PDAC patients, on the basis of the idea that PDAC is micro‐metastatic cancer in most cases since its diagnosis.^26^

One may speculate that the optimal ablation power setting for PDAC after chemotherapy may be an intermediate value of 30 W as a balance between the obtained necrosis and ablation times. Indeed, while in our tests there was some trend suggesting that the lowest 10 W value leads to a larger necrotic area, it requires a much longer time that may not be possible in vivo during EUS‐RFAs with multiple passages. On the other hand, at 50 W we obtained smaller necrosis limited by air bubbles due to increasing temperature, limiting heat diffusion, and providing a kind of “border” with the remaining surrounding neoplasia.

Other interesting results are a lack of correlation between US‐coagulative necrosis size at ablation end and actual histology size and the possibility to perform treatment with biliary stents on site. Ablation areas US‐detected were larger with respect to histology coagulative necrosis. Importantly, there was a very good agreement between the two pathologists in terms of necrotic area. Furthermore, RFA was performed with a metallic stent inside the biliary duct for the first time. This is a monopolar system and in clinical practice, having a ground pad outside the patient (and not into another closer point on the probe as in bipolar systems). The stent is usually removed during RFA and then replaced in patients with neoplastic jaundice. The present results of lack of system malfunctions or stops in the presence of metallic stents may warrant further investigation.

Finally, we investigated for the first time CT features that could be used to predict RFA response in PDAC. This is an intriguing issue, as it has been recently reported that CT features may predict photodynamic therapy response in PDAC.^27^ However, in our study there were no radiological features associated with treatment efficacy in terms of necrotic area. This specific issue may need further investigation with a larger sample size.

In conclusion, the present study suggests that ex‐vivo RFA with this system produces limited coagulative necrosis sizes of 80% regardless of the applied power. There are no clinical or radiological features able to predict treatment success and no clear advantage of a certain RFA power setting. Further rigorous studies are necessary to establish EUS‐RFA in‐vivo effects on locally advanced PDAC patients and to analyze long‐term oncological survival data to identify whether particular patients' subsets may preferentially benefit from this treatment modality.

## CONFLICT OF INTEREST

The authors declare no conflict of interest.

## FUNDING INFORMATION

None.

## Supporting information


**Figure S1**. Specimen ultrasound (US) evaluation before the ablation with an external US linear probe. The bowl containing scanty water and tumor specimen was connected to a radio frequency generator by grounding plates. RFA standard EUS needle was connected to the generator and manually inserted in the lesion under ultrasound control with a linear probe

**Figure S2**. Radiofrequency ablation system. A) needle, similar to an endoscopic ultrasound fine‐needle aspiration or biopsy needle with an electrode on the tip; B) peristaltic pump which can infuse electrode during the ablation with chilled solution, maximizing the ablation area; C) electrode on the distal needle tip, delivering the radiofrequency ablation; D) radiofrequency generator, with the possibility to monitor ablation parameters: power, time, impedance
