# Supplementary figures and images for: Ex‐vivo investigation of radiofrequency ablation in pancreatic adenocarcinoma after neoadjuvant chemotherapy
Source: DEN Open. 2022 Jul 14;3(1):e152. doi: 10.1002/deo2.152 (PMC9307734; doi:10.1002/deo2.152)

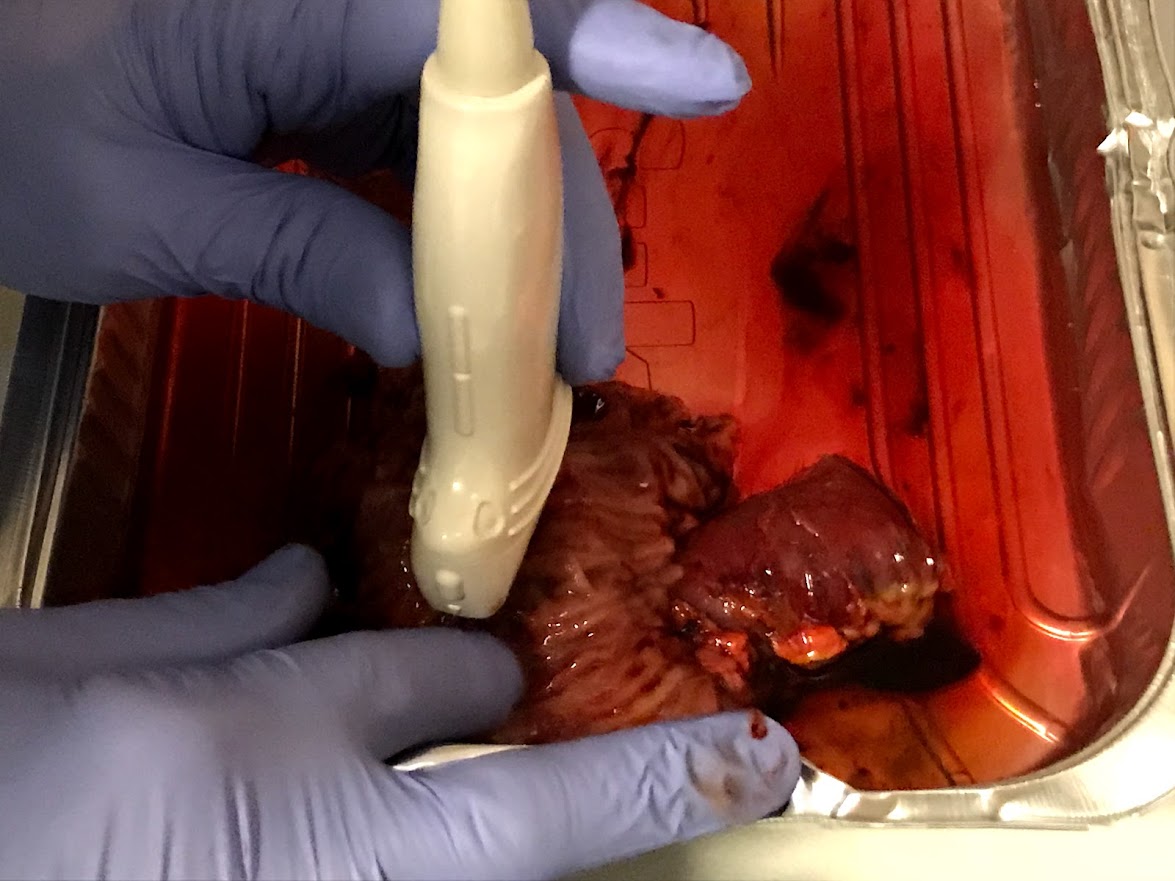

Supplement: Supplementary file 1 — Figure S1. Specimen ultrasound (US) evaluation before the ablation with an external US linear probe. The bowl containing scanty water and tumor specimen was connected to a radio frequency generator by grounding plates. RFA standard EUS needle was connected to the generator and manually inserted in the lesion under ultrasound control with a linear probe [file DEO2-3-e152-s001.jpg]

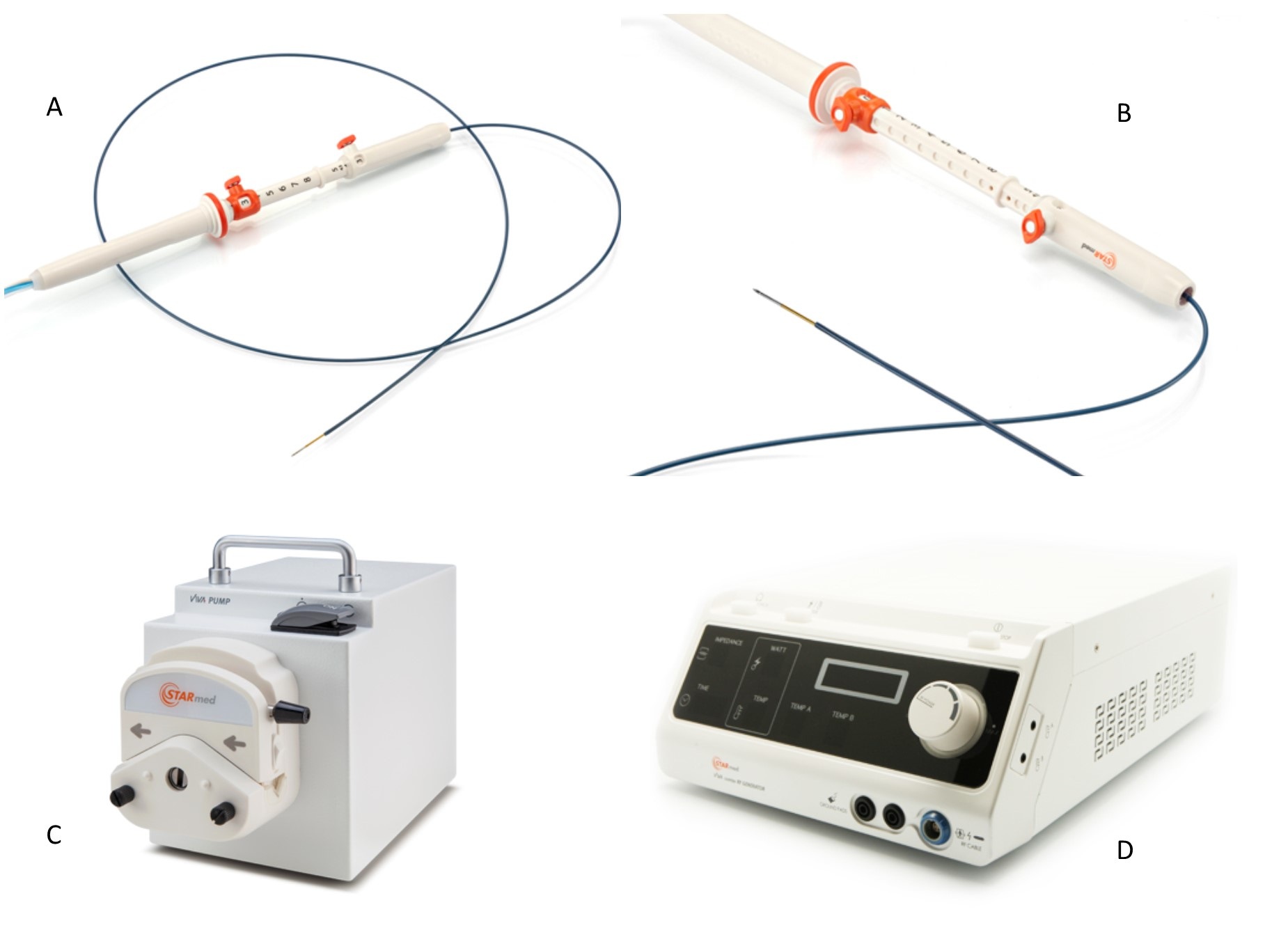

Supplement: Supplementary file 2 — Figure S2. Radiofrequency ablation system. A) needle, similar to an endoscopic ultrasound fine‐needle aspiration or biopsy needle with an electrode on the tip; B) peristaltic pump which can infuse electrode during the ablation with chilled solution, maximizing the ablation area; C) electrode on the distal needle tip, delivering the radiofrequency ablation; D) radiofrequency generator, with the possibility to monitor ablation parameters: power, time, impedance [file DEO2-3-e152-s002.jpg]
